# Transjugular Intrahepatic Portosystemic Shunt Placement: Effects on Nutritional Status in Cirrhotic Patients

**DOI:** 10.3390/jcm12227029

**Published:** 2023-11-10

**Authors:** Ilaria de Felice, Lorenzo Ridola, Oliviero Riggio, Jessica Faccioli, Silvia Nardelli, Stefania Gioia

**Affiliations:** Department of Translational and Precision Medicine, Sapienza University of Rome, 00185 Rome, Italy; ilaria.defelice@uniroma1.it (I.d.F.); lorenzo.ridola@uniroma1.it (L.R.); oliviero.riggio@uniroma1.it (O.R.); jessica.faccioli@uniroma1.it (J.F.); silvia.nardelli@uniroma1.it (S.N.)

**Keywords:** malnutrition, sarcopenia, myosteatosis, transjugular intrahepatic portosystemic shunt, portal hypertension

## Abstract

Malnutrition is a tangible complication of cirrhosis with portal hypertension with a prevalence of up to 50%. In particular, sarcopenia and myosteatosis, defined as the alteration in muscle quantity and quality, have a negative impact on the main complications of liver disease and are associated with higher mortality in patients with cirrhosis. Recently, alterations in adipose tissue have also been described in cirrhotic patients and they seem to influence the course of liver disease. Several pieces of evidence indicate that a transjugular intrahepatic portosystemic shunt (TIPS), placed for the treatment of refractory portal hypertension, can lead to a modification of body composition consisting in the improvement of the skeletal muscle index, myosteatosis, and an increase in subcutaneous fat. These modifications of the nutritional status, even more pronounced in sarcopenic patients before TIPS, have been associated with an amelioration of cognitive impairment after TIPS as well as with an increase in the survival rate. The aim of this paper is to provide an overview of the effects of TIPS placement on nutritional status in cirrhosis focusing on its pathophysiological mechanisms and its relationship with liver-related outcomes.

## 1. Criteria for Literature Selection

Clinical studies that assessed the modifications of body composition after TIPS placement in patients with cirrhosis were included. Studies that evaluated the prevalence and impact of malnutrition on liver disease-related outcomes in cirrhotic patients, and those that evaluated the pathophysiological mechanisms at the basis of the changes of nutritional alterations were included too. No language, publication date, or publication status restrictions were imposed. The studies were identified by searching electronic databases (PubMed and SCOPUS). The last search was run on 28 October 2023. Reference lists of all studies included in the present review were screened for potential additional eligible studies.

Two investigators (SG and IdF) searched the electronic databases by combining the following keywords: “sarcopenia”/“myosteatosis”/“body composition”/“body mass index”/“subcutaneous adipose tissue”/“fat”/“malnutrition”/“nutritional status”/“visceral adipose tissue” AND “cirrhosis”/“TIPS”/“transjugular intrahepatic portosystemic shunt”/“portal hypertension”/“cirrhotic patients”. Studies were excluded if the title and/or abstract showed that the articles did not meet the selection criteria of our review. For potentially eligible studies, or if the relevance of an article could not be excluded with certitude, we procured the full text. We excluded studies utilizing animal models. A total of twenty-eight papers were analyzed.

## 2. The Burden of Malnutrition in Cirrhosis: Definition, Epidemiology, and Impact

Nowadays, malnutrition is considered a tangible complication of cirrhosis and an independent factor of lower survival [1]. Certainly, it is more frequent in patients with decompensated cirrhotic disease (up to 50% of patients), but its prevalence is not neglectable even in patients with compensated disease (20%) [1].

The alterations of nutritional and metabolic status typically observed in cirrhosis are still potentially reversible conditions; however, they worsen liver disease progression and affect patients’ outcomes. In fact, malnutrition is independently associated with cirrhosis-related morbidity, mortality, and longer hospitalizations [2,3,4,5].

Sarcopenia is a major component and surrogate marker of protein malnutrition and is defined as the phenotypic manifestation of loss of muscle mass in cirrhosis [1,6]. It is likely attributed to the poor dietary intake, malabsorption, hypermetabolism, and decreased protein synthesis connected to anabolic resistance at the basis of cirrhosis [7,8]. Sarcopenia is an important and life-threatening complication of cirrhotic disease, repeatedly related to an increased risk of infection, hepatic encephalopathy, and length of hospitalization, both pre- and post-liver transplantation [9,10,11,12,13,14]. Moreover, sarcopenia may lead to adverse outcomes, such as physical disability, poor quality of life, waitlist mortality, and death [15].

The prevalence of sarcopenia in patients with cirrhosis ranges from 40 to 70% with a higher prevalence in men (54%) than in women (21%) [16]. It is assessed by the measurement at abdominal CT scan, considered the diagnostic gold standard, of the skeletal muscle index (SMI) which represents the cross-sectional measurement of muscle areas at the level of the third lumbar vertebra (L3) normalized to height [6,15]. The presence of sarcopenia is defined by validated and sex-based cutoffs: SMI cutoff of <39 cm/m^2^ in women, and <50 cm/m^2^ in men [1,16].

In recent years, even more studies have also investigated muscle quality, showing myosteatosis as a risk factor of poorer outcomes in cirrhotics. Myosteatosis refers to a gain of intermuscular and intramuscular fat [17]. Its presence is assessed at CT scan where a low radiodensity of psoas muscle at the level of the third lumbar vertebra (L3) is indicative of the adipose tissue disposition in the skeletal muscle. As well as sarcopenia, it is an independent prognostic factor for survival in cirrhotic patients. In fact, myosteatosis is associated with a higher prevalence of complications of liver disease, including hepatic encephalopathy (HE). It has been shown that the prevalence of myosteatosis is higher in patients with overt HE than in those without (70% vs. 45%) [11]. Furthermore, myosteatosis is also associated with a worsening of their physical condition and longer hospitalization in intensive care units [12,18].

Furthermore, with the availability of methods to study the quantitative alterations of the visceral adipose tissue (VAT) and subcutaneous adipose tissue (SAT), more recently, clinicians have also focused their attention on the possible role of fat in cirrhosis. These two types of adipose tissue differ in site, adipocyte size and function: SAT represents an accumulation of free fatty acids and triglycerides and has a pivotal role in the setting of lipid and glucose metabolism, while VAT is implicated in metabolic and cardiovascular disorders [18]. They have been shown to play a potential key role in some outcomes of cirrhosis [19]. In fact, it has been observed that the presence of high VAT is associated with an increasing risk of hepatocellular carcinoma in male patients and that low SAT may be a predictor of mortality in female patients with cirrhosis [19]. However, the clinical impact of adipose tissue alterations in cirrhotic patients are still poorly understood.

## 3. Nutritional Intervention in Cirrhosis

Due to the negative impact of protein malnutrition on liver-related outcomes, any integrated nutritional approach and exercise should be adopted to preserve physical status and to correct these alterations in cirrhotic patients [1,6,12,16].

All patients should be referred to routine screening for the prompt identification of malnutrition. When alterations of their nutritional status are identified, rapid interventions may be adopted. Firstly, nutritional management with personalized and balanced prescriptions should be guaranteed, reporting sufficient energy intake, optimal protein intake, and avoiding prolonged periods of fasting [20]. The European Association for the Study of the Liver (EASL) recommends a daily protein intake of 1.2–1.5 g/kg and an energy intake of at least 35 kcal/kg to reverse sarcopenia and prevent further loss of muscle mass.

However, in some cases short term enteral or parenteral nutrition should be proposed in association with branched chain amino acid (BCAA) supplements. Recent studies described the beneficial effects of BCAA on nutritional performance, such as event-free survival and quality of life [21,22]. In fact, leucine, isoleucine, and valine supplementation sustains protein synthesis, glucose, and lipid metabolism, decreasing oxidative stress in the hepatocytes; however, the timing of their administration and their dosage are now not properly clear [23].

Furthermore, the avoidance of prolonged fasting periods of more than six hours should be suggested; frequent meals, especially late-night snacks, could be a good option. Late-night light meals allow the improvement of fat free mass and total body protein stores, reducing lipid oxidation [24].

In addition to nutritional supplementation, physical activity and aerobic exercise are also determinants in preserving muscle mass and function. Beneficial effects have been reported in sporty cirrhotic patients who practiced routine physical activity. Aerobic and resistance exercise and training 3–5 times per week are recommended [1,12].

A multidisciplinary team composed of a gastroenterologist/hepatologist, health–behavior specialist, and dietician would be useful for patients in following each recommendation.

However, the correction of malnutrition in cirrhosis is still challenging and to date only limited evidence of the possibility to reverse the alterations of nutritional status has been shown [7,25,26].

## 4. Effects of TIPS on Nutritional Status in Cirrhosis and Possible Mechanisms

A transjugular intrahepatic portosystemic shunt (TIPS) consists of an artificial channel within the liver connecting the portal circulation to the systemic one, and was developed at the beginning of the nineties to reduce portal pressure and to manage complications related to portal pressure. Classic indications for TIPS placement, based on the European guidelines of decompensated cirrhosis and the Italian consensus on the positioning of TIPS, are refractory ascites and gastroesophageal variceal bleeding. Furthermore, other indications for TIPS exist, such as the failure of anticoagulants and mechanical revascularization in Budd Chiari Syndrome, bridge treatment in patients listed for liver transplant, or portal vein thrombosis [27,28,29,30,31,32]. However, hepatic encephalopathy represents a frequent complication of TIPS [33] affecting quality of life, requiring frequent hospitalizations, and sometimes the reduction of the caliber of the stent. Therefore, patient selection for TIPS requires careful assessment of risk factors for HE [34,35,36]. The search for the ideal candidate should take into account multiple parameters such as the history of HE and the presence of minimal HE, the comorbidities and age of the patient, liver and renal function, as well as the presence of alterations of nutritional status (i.e., sarcopenia and myosteatosis) [37].

The present review aimed to investigate the effects of TIPS placement on nutritional status and re-management of body composition in cirrhotic patients. All these new aspects are addressed in this review with a critical approach based on the literature review and clinical practice. Existing data on these eventual targets are limited, due to factors including few patients and a major part of studies being conducted in monocentric groups by retrospective studies. However, we will examine the relationship between TIPS and the improvement of muscle mass and adipose tissue in cirrhosis. Future research is needed to explore and validate the role of TIPS in these new scenarios.

In recent years, more and more evidence has described the positive effects of TIPS placement on clinical and physical status in cirrhotic patients. All these effects consisted of modifications of the whole-body composition [7,36], including modification in weight, muscle mass, and adipose tissue, leading to an improvement of nutritional status.

In their retrospective study on a series of 77 patients with cirrhosis, Pang et al. observed a significant increase in weight and body mass index (BMI) after TIPS (in men, the average weight and BMI increased by 2.5% and 2.5%, respectively, and in women, they increased by 4.4% and 4.3%, respectively) [38]. Interestingly, post-TIPS BMI was positively associated with pre-TIPS blood ammonia: the higher the blood ammonia levels before TIPS, the greater the increase in BMI. The authors hypothesized that high levels of ammonia could lead to more consumption of protein from muscle mass and that this depletion would be quickly restored after TIPS.

Furthermore, it has been shown that in normal weight cirrhotics, dry lean body mass as well as total body water improved after TIPS insertion [39]. However, these body modifications were not associated with significant changes in metabolic parameters.

In their prospective study on twenty-one cirrhotic patients submitted to TIPS for recurrent esophageal variceal bleeding (14 patients, five of whom also had ascites) and for refractory ascites (7 patients), Plauth et al. demonstrated a gain in muscle mass and body cell mass (BCM) after artificial portosystemic shunting confirming the actual amelioration of nutritional status and body composition [40].

### 4.1. Effect of TIPS on Muscle

Similarly, TIPS placement appeared to have a positive impact also on quantity and quality of muscle alterations in malnourished cirrhotic patients with a parallel positive impact on survival and some liver-related outcomes. In fact, several studies confirmed an amelioration of muscle mass quantity after TIPS with a reversal of sarcopenia in most of the patients. In a population of 179 cirrhotic patients submitted to TIPS placement and followed-up with repeated abdominal CT scans, Artru et al. [41] observed a significant increase in transversal right psoas muscle thickness at the umbilical level/height (TPMT) and total psoas muscle area (TPMA) at 1–3 months and 6 months after the intervention. There were no differences in the modifications of body composition between patients submitted to TIPS for the treatment of ascites and those submitted to TIPS for variceal bleeding. Studies assessing the presence and modifications of sarcopenia by the measurement of SMI showed a significant improvement of this parameter after TIPS [41,42,43,44].

Interestingly, all these modifications of muscle mass seemed to be more evident in patients with sarcopenia before TIPS, who experienced an improvement of SMI of approximately 8 cm^2^/m^2^ within 10–19 months after TIPS placement [45], higher than the improvement showed in non-sarcopenic patients. However, the reasons why the modifications of skeletal muscle mass occur only in a portion of patients are not completely understood. In order to investigate the factors influencing the improvement of sarcopenia after TIPS, Huang retrospectively analyzed the data of 111 cirrhotic patients with sarcopenia submitted to TIPS. Six months after TIPS, SMI increased significantly from the basal value both in women and in men. Using a multivariate logistic regression analysis, pre-TIPS SMI (odds ratio [OR], 0.93; 95% CI, 0.87–0.99; *p* = 0.031) and change in portal pressure gradient (OR, 1.13; 95% CI, 1.03–1.24; *p* = 0.009) were found to be independent risk factors for the improvement of SMI after TIPS [46].

March et al. evaluated changes in skeletal muscle mass after TIPS implantation using both the psoas muscle index (PMI) and the SMI and compared the power of PMI and SMI as measures of sarcopenia [47]. First, the authors confirmed the positive impact of TIPS placement on muscle mass. According to SMI and PMI cutoffs, most of the 52 patients included in the study were defined as sarcopenic at baseline. After a median follow-up of 16.5 months from TIPS insertion, sarcopenia reversed in many patients, decreasing from 85% to 70% (with SMI cutoff) and from 92% to 68% (with PMI cutoff). On the contrary, no significant modifications in SMI and/or PMI were observed in non-sarcopenic patients at baseline. This result is in line with previous findings [45]. While no correlation between SMI and PMI before TIPS was detected, a positive correlation between these two parameters after TIPS was shown. Interestingly, this is one of the few studies that assessed muscle mass modifications after TIPS considering the etiology of cirrhosis. In fact, patients with alcoholic etiology of cirrhosis showed significant differences in the psoas muscle area (PMA) and PMI after TIPS compared to patients with all other etiologies, with no significant differences before the TIPS procedure. Patients with alcoholic liver disease experienced a greater change in PMA than the other patients. Finally, authors investigated a possible correlation of the muscle indices with the MELD and the Freiburg index of post-TIPS survival (FIPS). In more detail, patients were stratified according to FIPS, which is a score that is able to predict post-TIPS survival. By analyzing similar parameters, the two scores are strongly correlated. However, while a strong and negative correlation between the MELD score after TIPS and PMI after TIPS was shown, no correlation between FIPS and muscle parameters before and after TIPS was observed (probably due to the limited sample size).

Contrarily, Xiong et al. assessed whether sarcopenia may have added value on the existing risk scores used to predict survival after TIPS. In their cohort of 386 patients affected by cirrhosis undergoing TIPS, five existing scores for the prediction of short- and long-term mortality after TIPS were compared (Child-Pugh, MELD, MELD-Na, MELD 3.0, and FIPS) and among them the FIPS was shown to be the most powerful. Interestingly, FIPS significantly correlated with the presence and severity of sarcopenia before TIPS and with its reversal after TIPS. Moreover, the addition of sarcopenia to this score improved survival prediction and risk stratification [48].

Also, the quality of muscle mass was modified after TIPS with a significant reduction of myosteatosis [49]. Moreover, the improvement of sarcopenia and myosteatosis has been associated with better survival [41,50] and to an amelioration of cognitive impairment after TIPS, consisting of a reduced prevalence of minimal hepatic encephalopathy and a lower number of episodes of post-TIPS hepatic encephalopathy. The last observation again confirms the key role of muscle in the pathogenesis of HE.

Recently, the impact of TIPS on muscle function, performance and frailty has been evaluated. Surprisingly, the muscle increase observed after TIPS as well as that of Insulin Growth Factor 1 (IGF-1), a marker of muscle anabolism, was not accompanied by the improvement in muscle function measured by handgrip strength, frailty (liver frailty index), or physical performance. Hey et al. speculated that it was probably related to impairment in muscle quality and the effects of hyperammonemia on muscle contractile function [51].

### 4.2. Effect of TIPS on Adipose Tissue

As previously explained, more recently, the attention of clinicians and researchers has also focused on the alterations and properties of fat, specifically of subcutaneous and visceral fat. In fact, parallel to the modifications of muscle composition, the characteristics and quantity of adipose tissue also changed after TIPS. In more detail, Artru et al. showed an increase in subcutaneous fat and a decrease in visceral fat within the first 6 months after TIPS placement [41]. Gioia et al. similarly observed that after a mean follow-up of 19 ± 15 months after TIPS placement, the visceral adipose tissue index (VATI) significantly reduced and the subcutaneous adipose tissue index (SATI) significantly increased in the majority of patients, independent of liver function [43]. Again, patients who experienced an improvement in subcutaneous adipose tissue had a lower number of post-TIPS HE than patients without an improvement in subcutaneous adipose tissue. A correlation between the differences of subcutaneous fat (delta-SATI) and of ammonia before and after TIPS has been observed, probably suggesting a role of subcutaneous adipose tissue on ammonia handling and on the reduction in ammonia levels. The possible key role of adipose tissue on the pathogenesis of hepatic encephalopathy is further suggested by the results of the study conducted by Wang who showed that females with low SATI (<70.05 cm^2^/m^2^) (OR 10.55; 95% CI 2.36–46.23; *p* = 0.002) and male patients with low VATI (<53.52 cm^2^/m^2^) (OR 6.44; 95% CI 1.72–23.59; *p* = 0.006) had a higher risk of post-TIPS HE. In fact, SATI and VATI were confirmed to be predictors of hepatic encephalopathy after TIPS in a multivariate analysis [52].

Interestingly, Gioia et al. described that changes in muscular and adipose tissue were more evident in cirrhotic patients with ascites than in those without ascites [43]. The studied population included 35 cirrhotic patients, of whom 19 were submitted to TIPS for refractory ascites and 16 for variceal bleeding. Moreover, seven out the latter patients also had ascites. In the 23 patients who experienced an increase in SMI values, the initial SMI value was significantly lower and the prevalence of ascites before TIPS placement was significantly higher (86%) than in the 13 patients without an increase (54%). Similarly, the presence of ascites was more frequent in patients with an increase in SATI compared to those without (82% vs. 43%).

This occurs probably because ascites increases energy expenditure and could play a key role in protein–energy malnutrition. TIPS placed for refractory ascites not only contributes to better quality of life for decreasing abdominal discomfort, but also to a reduction of basal energy expenditure, directly connected to the amelioration of malnutrition. In Table 1 the main results of published studies on the modifications of body composition after TIPS in patients affected by cirrhosis are summarized.

### 4.3. Pathophysiological Mechanisms Involved in the Modifications of Body Composition after TIPS

Several hypotheses have been debated about the possible pathophysiological mechanisms at the basis of the nutritional targets obtained in patients receiving TIPS. Probably, one relevant consideration is that the improvement in metabolic and nutritional status is strictly associated with the reduction of portal hypertension obtained with TIPS placement [39]. In fact, portal hypertension damages the intestinal barrier integrity causing intestinal permeability and the consequent gut bacterial translocation and systemic diffusion of lipopolysaccharides (LPS) and other pro-inflammatory molecules, including interleukin-1β, interleukin-6, and Tumor Necrosis Factor α (TNF-α). This inflammatory imbalance promotes a catabolic effect in cirrhotic patients, also known as anabolic resistance, leading to protein mass wasting [5,7,39]. On the other hand, the reduction of portal pressure by TIPS also reduces protein losing enteropathy, gastroparesis, and small bowel dysmotility. Normal food absorption in the gastrointestinal tract can then directly be obtained leading to an increase in the patient’s energy intake [53]. It has been shown in this way, that total resting energy expenditure increases after TIPS and an additional energy demand could be necessary for the growth of muscle mass. As proposed by some authors, protein synthesis requires a 30% increase in energy and protein intake 6 months after TIPS positioning [7,36,38,40,41].

In actuality, the reset of portal hypertension not only guarantees restoration of anabolic functions, but also has a place in the management of metabolic and hormonal mechanisms, such as reducing insulin resistance [38]. Decreased insulin resistance has an optimal impact on the body’s glucose metabolism as well as on increasing the use of glucose in peripheral tissues and reducing the consumption of body proteins [4]. The correction of disorders related to portal hypertension results in a reversal in the hypermetabolism of cirrhosis, where various regulatory signaling proteins take part. For example, a reduction in plasma leptin concentration or an increase in circulating Insulin Growth Factor 1 (IGF-1) promptly stimulates protein synthesis and locks proteolysis in skeletal muscle, as well as reduces muscle myostatin, a transforming growth factor β (TGF β) superfamily member that is responsible for reduced skeletal muscle mass [7,50]. Furthermore, it has been demonstrated that noradrenaline concentration, as well as that of aldosterone and plasma renin activity, normalized 4–6 months after TIPS. This leads to a reversal of the hyperdynamic circulation typical of cirrhosis and most evident in patients with ascites [54,55]. Moreover, the reduction of the levels of noradrenaline (725 vs. 304 pg/mL) may have an impact on the nutritional and metabolic modifications after TIPS. In fact, catecholamines are involved in glucose metabolism, being responsible for the maintenance of normal blood glucose levels by stimulating glucagon release, glycogenolysis, and food consumption, and by inhibiting insulin release.

Tomsen et al. also examined an advanced shift in the pattern of particular types of adipokines in patients with cirrhosis and showed that adiponectin increased after TIPS while Retinol binding Protein 4 (RBP4) decreased. Adiponectin enhances muscle fat oxidation and glucose transport, increasing appetite and weight gain; on the other hand, RPB4 is a negative regulator of insulin action, therefore its reduction is associated with improved whole-body metabolism, avoiding features of metabolic syndrome [2,43].

A confirmation of the role of portal hypertension in determining muscle wasting may be the demonstration of the presence of sarcopenia in patients with non-cirrhotic portal hypertension (NCPH), who have marked portal hypertension with only mild liver damage. In fact, in the retrospective study designed by Lattanzi et al., patients affected by chronic portal vein thrombosis and idiopathic non-cirrhotic portal hypertension had a prevalence of sarcopenia of 35% and 38%, respectively, and the prevalence of sarcopenia in a control group of cirrhotic patients was of 40% [56]. Furthermore, when cirrhotic patients were stratified according to liver disease severity, the prevalence of sarcopenia in patients with NCPH was similar to patients with compensated cirrhosis (MELD < 15), while that of cirrhotic patients with MELD ≥ 15 was slightly, but not significantly, higher.

Interestingly, sarcopenic patients with NCPH presented a superior rate of rebleeding requiring TIPS in comparison with the non-sarcopenic ones, suggesting that sarcopenic patients seemed to be more prompt in developing complications related to portal hypertension.

These considerations bring out the prognostic role of sarcopenia and real improvements in nutritional status after the correction of portal hypertension.

## 5. Conclusions and Future Perspectives

**Figure 1:**
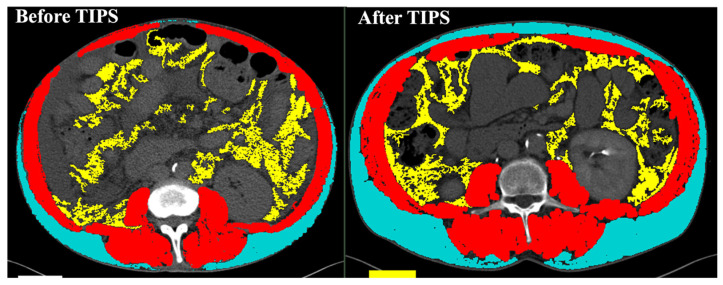
Modifications of muscle mass and adipose tissue after TIPS placement. Contrast-enhanced abdominal CT scan of a 72 year old male cirrhotic patient submitted to TIPS for treatment of refractory ascites. Six months after TIPS placement, the skeletal muscle area (red) increased from 15,976 cm^2^ to 19,266 cm^2^ with an increase in SMI from 49.3 cm^2^/m^2^ to 59.4 cm^2^/m^2^; parallelly, subcutaneous adipose tissue (SAT) (light blue) increased from 5649 cm^2^ to 13,248 cm^2^ with an increase in SATI from 17.4 cm^2^/m^2^ to 40 cm^2^/m^2^. Visceral adipose tissue (VAT) (yellow) decreased from 9202 cm^2^ to 7809 cm^2^ with a decrease in VATI from 28 cm^2^/m^2^ to 24 cm^2^/m^2^.

In conclusion, we described how TIPS placed for typical complications of portal hypertension results in the amelioration of body composition (Table 1) (Figure 1). All these observations may change or at least lead clinicians to reconsider the selection of patients to submit to TIPS. In fact, at the moment, sarcopenia is considered a relative contraindication for TIPS placement, due to the higher risk of mortality [57] and overt hepatic encephalopathy after the procedure as shown in sarcopenic patients before TIPS. Furthermore, it has also been shown that a lower quantity of intra- and intermuscular adipose tissue, a condition known as adipopenia, in relation to total muscle and skeletal volume was associated with higher mortality rates one year after TIPS placement [58].

On the other side, TIPS could be a good chance to reverse malnutrition with a positive effect on the prognosis and complications of liver disease. This goal takes on even more importance in relation to the fact that to date, there are few nutritional interventions that effectively allow the correction of malnutrition in cirrhotic patients. Several limitations still exist due to the small sample size of studies exploring the modifications of body composition induced by TIPS placement, and to different potential biases such as the various techniques used to evaluate muscle alteration, the selection of patients, or the different timings to evaluate changes after TIPS. However, future strategies could define the therapeutic role of TIPS in sarcopenia as well as for myosteatosis and adipose tissue alterations in cirrhosis.

## Figures and Tables

**Table 1:** Published studies on the modifications of body composition after TIPS in patients affected by cirrhosis.

Reference	No. of Patients	TIPS Indication	Follow-Up after TIPS	Parameters	Modifications
Allard et al. (2001) [36]	14	RA (14)	12 M	Weight, dry weight, FM, TBF, MRR, F10/F30	Improvement in dry weight and FM
Plauth et al. (2004) [40]	21	RA (7), VB (14)	12 M	BMI, BCM, weight	Improvement in weight, BMI
Montomoli et al. (2010) [39]	21	RA (12), VB (7), both (2)	13.5 M	Dry lean mass, BMI, and FM	Significant increase in dry lean body in under/normal weight patients
Holland-Fischer et al. (2010) [4]	11	RA (7), VB and RA (4)	6 M	Weight, BCM, BMI, FM	Significant increase in weight, BCM, BMI
Thomsen et al. (2012) [2]	25	RA (17), VB (5), both (3)	6 M	Weight, BCM, BMI, FM, adiponectin, leptin, RBP4	Significant increase in anabolic stimulus and BCM
Tsien et al. (2013) [50]	57	RA (41), VB (14), both (2)	6 M	BMI, SMA, VAT, SAT	Increase in SMA, decrease in SAT
Gioia et al. (2019) [42]	27	RA (15), VB (12)	9.8 M	SMI, MA	Significant increase in SMI and MA
Artru et al. (2020) [41]	179	RA (94), VB (85)	6 M	SAT, SMI, TPMA, VAT	Significant increase in SAT, SMI e TPMA and significant decrease in VAT
Pang et al. (2021) [38]	77	RA (NS), VB (NS)	36 M	BMI, weight	Significant increase in BMI and weight, physical status
Gioia et al. (2021) [43]	35	RA (19), VB (16)	19 M	SMI, MA, SATI, VATI	Significant increase in SMI, MA and SATI, significant decrease in VATI
Liu et al. (2022) [44]	224	RA (31), VB (193)	12 M	SMA, SMI, SFA, SFT, weight, BMI	Increase in SMA, SMI, SFA, and SFT in sarcopenic patients. Increase in weight and BMI in sarcopenic patients without ascites.
Hey et al. (2023) [51]	12	/	6 M	SMA, SFA, intermuscular adipose tissue, MA, VAT, liver frailty index, handgrip	Significant increase in SMA, SFA, intermuscular adipose tissue. No improvement in MA, VAT, liver frailty index, handgrip.
Huang et al. (2023) [46]	111	/	6 M	SMI	Increase in SMI
March et al. (2023) [47]	52	RA (39), VB (11), other (2)	16 M	PMA, TMA, PMI, SMI	Increase in TMA, PMA, SMI, and PMI

List of abbreviations: RA (refractory ascites), FM (fat mass), TBF (total body fat), MRR (muscle relaxation rate), F10/F30 (force of adductor pollicis expressed as a %), VB (variceal bleeding), BMI (body mass index), BCM (body cell mass), RBP4 (retinol binding protein 4), SMA (skeletal muscle area), VAT (visceral adipose tissue), SAT (subcutaneous adipose tissue), SMI (skeletal muscle index), MA (muscle attenuation), TPMA (total psoas muscle area), NS (not specified), SATI (subcutaneous adipose tissue index), VATI (visceral adipose tissue index), SFA (subcutaneous fat area), SFT (subcutaneous fat thickness), PMA (psoas muscle area), TMA (total muscle area), PMI (psoas muscle index).

## Data Availability

Data sharing is not applicable to this article as no new data were created or analyzed in this study.

## Abbreviations

Computed tomography (CT); skeletal muscle index (SMI); third lumbar vertebra (L3); hepatic encephalopathy (HE); visceral adipose tissue (VAT); subcutaneous adipose tissue (SAT); European Association for the Study of the Liver (EASL); branched chain amino acid (BCAA); transjugular intrahepatic portosystemic shunt (TIPS); body mass index (BMI); body cell mass (BCM); transversal psoas muscle thickness (TPMT); total psoas muscle area (TPMA); odds ratio (OR); confidence interval (CI); psoas muscle index (PMI); psoas muscle area (PMA); Model for End-Stage Liver Disease (MELD); Freiburg index of post-TIPS survival (FIPS); Insulin Growth Factor 1 (IGF-1); visceral adipose tissue index (VATI); subcutaneous adipose tissue index (SATI); lipopolysaccharides (LPS); tumor necrosis factor α (TNF-α); transforming growth factor β (TGF-β); retinol binding protein 4 (RBP4); non-cirrhotic portal hypertension (NCPH).
